# Multifunctional green synthesized Cu–Al layered double hydroxide (LDH) nanoparticles: anti-cancer and antibacterial activities

**DOI:** 10.1038/s41598-022-13431-7

**Published:** 2022-06-08

**Authors:** Mahsa Kiani, Mojtaba Bagherzadeh, Amir Mohammad Ghadiri, Pooyan Makvandi, Navid Rabiee

**Affiliations:** 1 Department of Chemistry, Sharif University of Technology, Tehran, Iran; 2 Center for Materials Interfaces, Istituto Italiano di Tecnologia, viale Rinaldo Piaggio 34, 56025 Pontedera, Pisa Italy; 3 School of Engineering, Macquarie University, Sydney, NSW 2109 Australia

**Keywords:** Biotechnology, Diseases, Medical research

## Abstract

Doxorubicin (DOX) is a potent anti-cancer agent and there have been attempts in developing nanostructures for its delivery to tumor cells. The nanoparticles promote cytotoxicity of DOX against tumor cells and in turn, they reduce adverse impacts on normal cells. The safety profile of nanostructures is an important topic and recently, the green synthesis of nanoparticles has obtained much attention for the preparation of biocompatible carriers. In the present study, we prepared layered double hydroxide (LDH) nanostructures for doxorubicin (DOX) delivery. The Cu–Al LDH nanoparticles were synthesized by combining Cu(NO_3_)_2_·3H_2_O and Al(NO_3_)_3_·9H_2_O, and then, autoclave at 110. The green modification of LDH nanoparticles with *Plantago ovata* (PO) was performed and finally, DOX was loaded onto nanostructures. The FTIR, XRD, and FESEM were employed for the characterization of LDH nanoparticles, confirming their proper synthesis. The drug release study revealed the pH-sensitive release of DOX (highest release at pH 5.5) and prolonged DOX release due to PO modification. Furthermore, MTT assay revealed improved biocompatibility of Cu–Al LDH nanostructures upon PO modification and showed controlled and low cytotoxicity towards a wide range of cell lines. The CLSM demonstrated cellular uptake of nanoparticles, both in the HEK-293 and MCF-7 cell lines; however, the results were showed promising cellular internalizations to the HEK-293 rather than MCF-7 cells. The in vivo experiment highlighted the normal histopathological structure of kidneys and no side effects of nanoparticles, further confirming their safety profile and potential as promising nano-scale delivery systems. Finally, antibacterial test revealed toxicity of PO-modified Cu–Al LDH nanoparticles against Gram-positive and -negative bacteria.

## Introduction

Cancer is one of the leading causes of death after cardiovascular diseases and various factors such as gene mutations, age and family history among others, can result in tumor imitation and development. The most common therapeutic modality for cancer is chemotherapy. However, complicated interactions occurring in tumor microenvironment and ability of cancer cells in proliferation and switching among molecular pathways to ensure their survival, have resulted in drug resistance^1–4^. Doxorubicin (DOX) is extensively utilized for cancer chemotherapy and it is capable of preventing DNA replication by inhibiting activity of topoisomerase enzymes to suppress cell cycle progression and direct cancers towards cell death^5–9^. However, various mechanisms such as efflux of DOX by P-glycoprotein from cancer cells, Bcl-2 upregulation, apoptosis inhibition and abnormal expression of genetic and epigenetic factors have resulted in DOX resistance^10^. Hence, studies have focused on designing nanoscale delivery systems for DOX to boost its tumor-suppressor activity. In a recent effort, MOF-5/MXene nanoarchitectures were modified with chitosan and alginate and then, DOX and CRISPR were loaded in nanoparticles. This co-delivery of gene and drug exerts synergistic impact in suppressing tumor progression and DOX-loaded MOF-5/MXene nanoparticles effectively reduced viability of cancer cells such as HEK-293, HepG2 and HeLa cells^11^. In another experiment, organic–inorganic carbon-based nanomaterials based on reduced graphene oxide and multi-walled carbon nanotubes were prepared to co-delivery DOX and CRISPR. These nanostructures provided prolonged release of DOX and were able to diminish tumor cell viability (HEK-293) up to 91.4%^12^. A recent experiment has developed ATP-responsive mesoporous silica nanoparticles for DOX delivery. This nanostructure released DOX in response to ATP levels and provided sustained release, leading to a decrease in viability of breast and colon cancer cells^13^. Another study developed hydroxyapatite carriers for DOX delivery and more release occurred in pH 5 (36%) compared to pH 7.4 (28%). These DOX-loaded nanostructures significantly reduced viability of osteosarcoma in vitro and in vivo^14^. Therefore, nanostructures provide new hopes in preventing drug resistance and enhancing potential of chemotherapeutic agents in cancer therapy^15^.

The layered double hydroxide (LDH) are layered nanostructures and anionic clays with hydrotalcite crystal structure that can be formed by two metal ions including a divalent and a trivalent metal ion, a hydroxyl group, water molecule and interlayer anion^16^. The general formulation of LDH is [M^2+^_1−*x*_M^3+^_*x*_(OH)_2_]^*x*+^[A^*n*−^]_*x*/*n*_·*m*H_2_O that M^2+^ and M^3+^ are divalent and trivalent ions, respectively, and x is the molar ratio that is at range of 020–0.33 to prevent unfavorable phase formation^17^. The LDH nanoparticles have their own application in engineering fields such as water treatment and they can also be used for heat transferring. Furthermore, they are promising options for photochemistry, adsorption, catalysis and molecular recognition^18–23^. Recently, LDH nanostructures have opened their way in biomedical field due to their unique layered structure and interlayer anionic exchange capacity^24^. One of the most important application of LDH nanoparticles in drug delivery that a certain compound can be embedded into LDHs via interlayer anion exchange. LDHs have a variety of good characteristics including high drug loading ability, large surface area, high stability and capacity in anion exchange, making them superior and better nanostructures for drug delivery compared to other kinds such as polymeric nanoparticles^25–27^. More importantly, LDHs are ideal candidates for cancer drug delivery due to dissolved bulk layer of LDHs at pH 5.0 (near to tumor microenvironment pH) that provides pH-sensitive release at cancer site^24^. It was shown that Cu-containing LDH nanoparticles escape from uptake by macrophages at physiological pH 7.4, while they internalize into cancer cells at pH 6.8 due to detachment of coated polymer layer^28^. Another experiment loaded indocyanine green in Cu-containing LDH nanoparticles to provide chemotherapy and photothermal therapy for breast cancer ablation^29^. In another effort, Mn–Al-containing LDH nanoparticles were prepared and loaded with fluorouracil that can be considered as promising agents for cancer treatment (anti-tumor drug delivery) and diagnosis (manganese as a contrast agent for MRI)^30^.

*Plantago ovata* (PO) is a traditional herbal medicine and has polysaccharides as bioactive components. PO is a naturally occurring compound that possesses benefits including affordability, sustainability, safety profile and accessibility^31^. The PO was first used for purpose of wound healing^32^. Further investigation demonstrated that extract of PO has therapeutic activities including antioxidant, anti-inflammatory, immuno-modulatory and analgesic, among others^33^. There have been efforts in synthesis of nanoparticles with PO to improve their characteristics. An experiment synthesized silver nanoparticles with PO and resulting nanoparticles demonstrated particle size of 13 nm with antibacterial activity (*Staphylococcus aureus*)^34^. Another study synthesized silver nanoparticles from PO with antibacterial activity against *Pseudomonas aeruginosa*^35^.

**Scheme 1:**
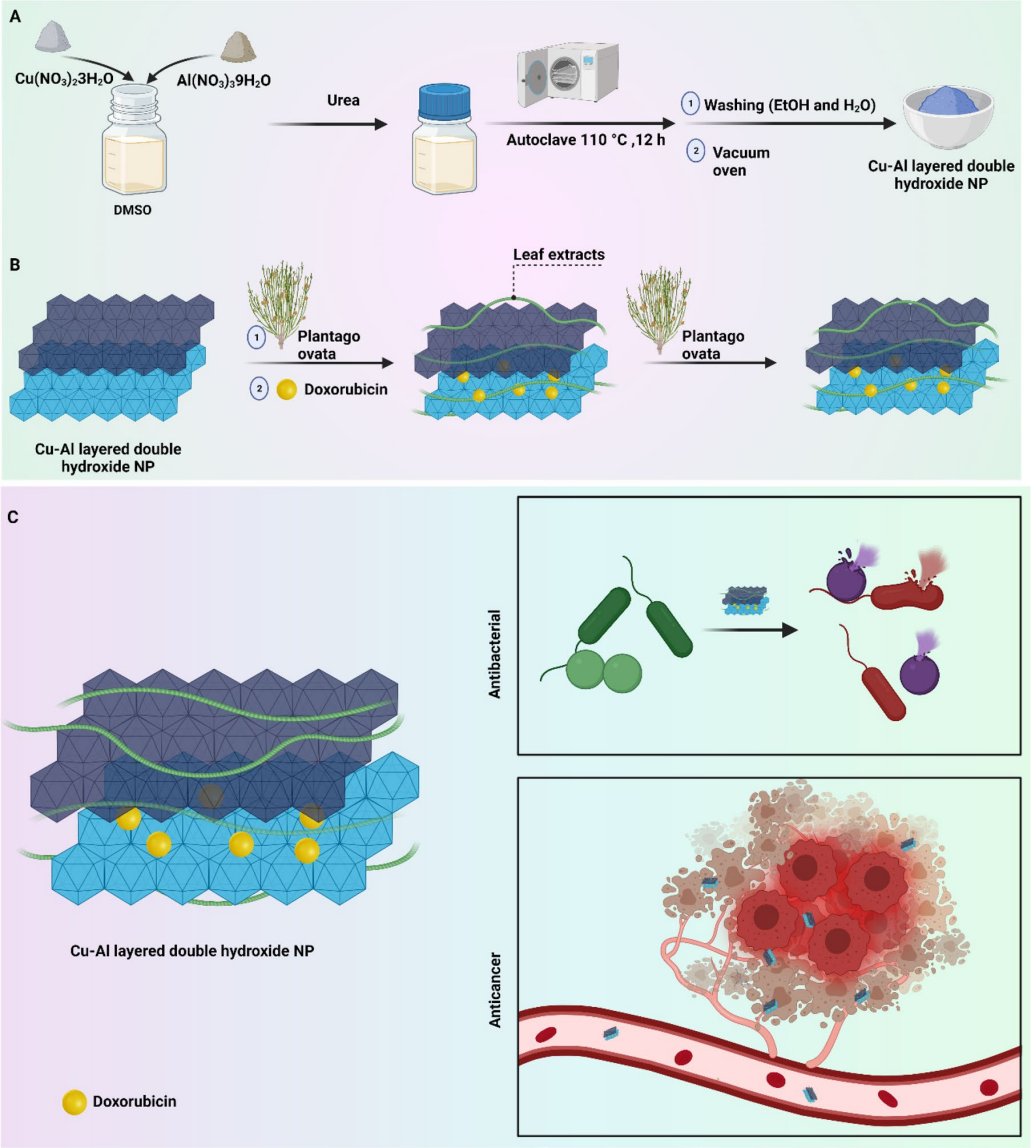
(**A**) Schematic illustration of synthesis of CuAl LDH. (**B**) Modification of CuAl LDH with *Plantago ovata* extracts and encapsulation of doxorubicin. (**C**) Schematic presentation of potential application of multifunctional green synthesized Cu–Al layered double hydroxide (LDH) nanoparticles for anti-cancer and antibacterial activities.

In the present study, prepared Cu–Al LDH nanoparticles were modified by PO extract as a naturally occurring compound and then, DOX was loaded as an anti-cancer agent in this nanoformulation. The Cu^2+^ and Al^3+^ as divalent and trivalent ions were applied, respectively. Various techniques including FTIR, XRD and FESEM were used for characterization of nanostructures. Drug release was examined in pH 4.5, 5.5 and 7.2 and MTT assay was used to evaluate anti-tumor activity of prepared nanostructures. The in vivo experiment was performed to further confirm potential of LDH nanoparticles in cancer suppression. Furthermore, PO-modified Cu–Al LDH nanoparticles demonstrated cytotoxicity against Gram-positive and -negative bacteria. Scheme 1 provides a summary of nanoparticle preparation.

## Materials and methods

### Synthesis of Cu–Al LDH

Briefly, 9 mmol of Cu(NO_3_)_2_·3H_2_O and 4.5 mmol of Al(NO_3_)_3_·9H_2_O and water (45 mL) along with DMSO (15 mL) as the solvents stirred for about 30 min, and then poured into the autoclave (Teflon-lined stainless-steel autoclave), urea (0.2 M; 60 mL) added to the autoclave and heated to 110 °C for 12 h. Then the autoclave was held at room temperature to cool down, and the Cu–Al LDH product was washed several times with ethanol and deionized water and drying in a vacuum oven at 80 °C overnight. It should be noted that no attention was paid to control the chemical and physical characteristics in this synthesis method.

### Modification of Cu–Al LDH with *Plantago ovata* extracts

In order to modify the surface and even the bulk porosity of the Cu–Al LDH with the PO leaf extracts, 1 g of the synthesized Cu–Al LDH was transferred to a beaker, and stirred for 5 h along with 10 mL of the leaf extracts. The obtained nanomaterial names as Cu–Al LDH@PO.

### Molecule modification on the Cu–Al LDH@PO

The prepared green LDH, Cu–Al LDH@PO was dispersed in the ultrapure water and stirred for 10 min. In order to modify the surface of the LDH, 10 mg of each molecule was added to the suspension and stirred for about 20 min at room temperature. The molecules were adsorbed on the surface of the nanocomposite via π–π interactions between the capped leaf extracts and the π-ring of the molecules, and through the potential hydrogen and van der Waals bonds/interactions between them. For ease of recognition, synthesized nanomaterial, the nano-substrate of Cu–Al LDH@PO is denoted “**A”** in the rest of this manuscript). Also, **A**-4-(2-(4-(3-carboxypropanamido)benzoyl)hydrazineyl)-4-oxobutanoic acid, **A**-4-fluoro-*N*-(4-(2-(4-fluorobenzoyl)hydrazine-1-carbonyl)phenyl)benzamide, **A**-4-methoxy-*N*-(4-(2-(4-methoxybenzoyl)hydrazine-1-carbonyl)phenyl)benzamide, **A**-4-nitro-*N*-(4-(2-(4-nitrobenzoyl)hydrazine-1-carbonyl)phenyl)benzamide and **A**-*N*-(4-(2-(phenylsulfonyl)hydrazine-1-carbonyl)phenyl)benzenesulfonamide are labeled **A-3a**, **A-3b**, **A-3c**, **A-3d** and **A-3e**, respectively, throughout the article.

### Drug loading onto nanoparticles

LDH nanostructures and DOX were incubated for 24 h at 4 °C and then, placed in a shaker at three ratios (one to one, two to one, and one to two) with the purpose of loading the DOX on the nanostructures. The samples were centrifuged at 4000 rpm for 5 min after 12 h to precipitate the DOX associated with the nanostructures^36,37^. The loading efficiency of drug can be calculated using the following equation:

1 $$EE\left(\text{\%}\right)=\frac{{\text{Total amount of DOX}}-{\text{Free DOX in precipitant}}}{\text{Total amount of DOX}}\times 100.$$

### Drug release evaluation

DOX release from modified and non-modified LDH nanostructures in phosphate-buffered saline (PBS) at 37 °C was measured during an 8-day period. The DOX-loaded LDH nanostructures were kept in a dialysis bag (MWCO: 12 kDa) containing a PBS solution for various lengths of time and then, centrifuged to calculate DOX release^38,39^. The DOX release concentration from LDH nanostructures was evaluated using absorbance at 450 nm using UV–Vis. The following equation is used in order to calculate the drug release ratio at the initial DOX level:

2 $$\text{DL}\left(\text{\%}\right)=\frac{\text{Total amount of DOX}-\text{Free DOX in precipitant}}{\text{Mass of final formulation}}\times 100.$$

### In vitro cytotoxicity assay

The MTT assay was used to evaluate cytotoxicity of nanostructures. For this purpose, four cell lines including HEK-293 (human embryonic kidney), PC12 (neuronal cells), HT-29 (colon adenocarcinoma) and MCF-7 (breast cancer) cell lines were chosen and incubated with modified and non-modified LDH nanostructures for 24 h and 48 h time intervals. The nanostructure-free medium was used as control experiment. For each experiment, we performed six replications. An ELISA reader was utilized in order to read the wells’ optical absorption at 492 nm wavelength.

3 $$\text{Cell viability}\left(\text{\%}\right)=\frac{\text{OD}492\text{ of sample}}{\text{OD}492\text{ of control}}\times 100.$$

## Results and discussion

### Synthesis and characterization

**Figure 1:**
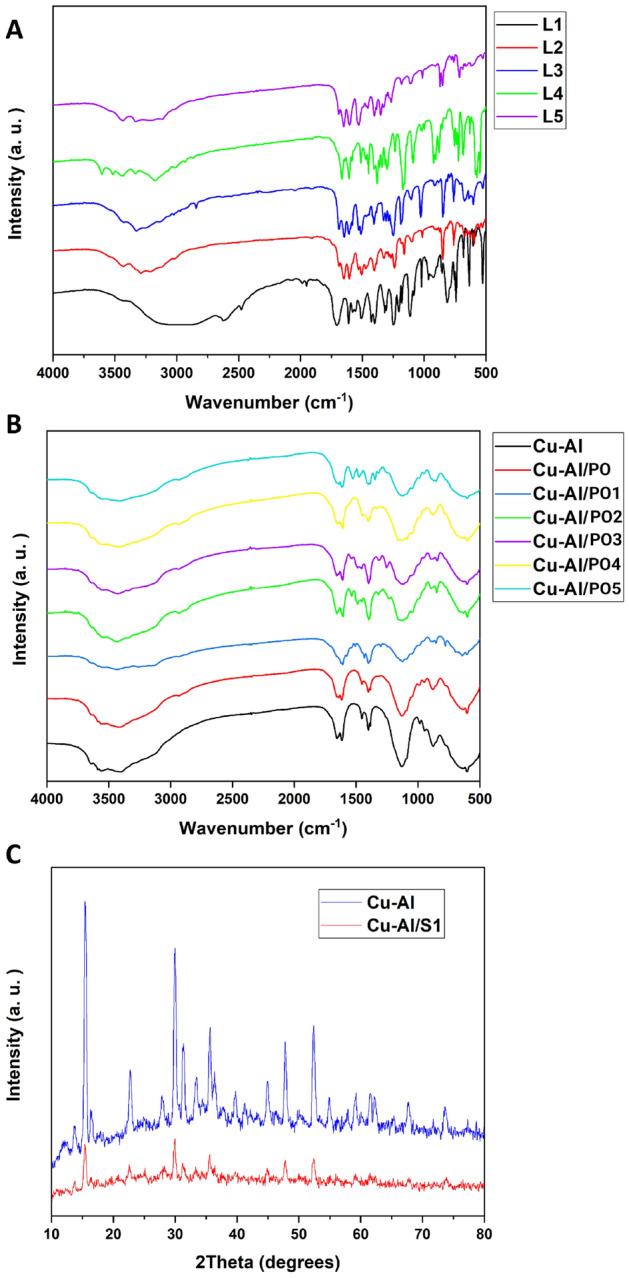
FTIR spectra of the synthesized nanomaterials (**A,B**). XRD results of the synthesized nanomaterials (**C**). S1 in part (**C**) stands for one of the PO’s.

**Figure 2:**
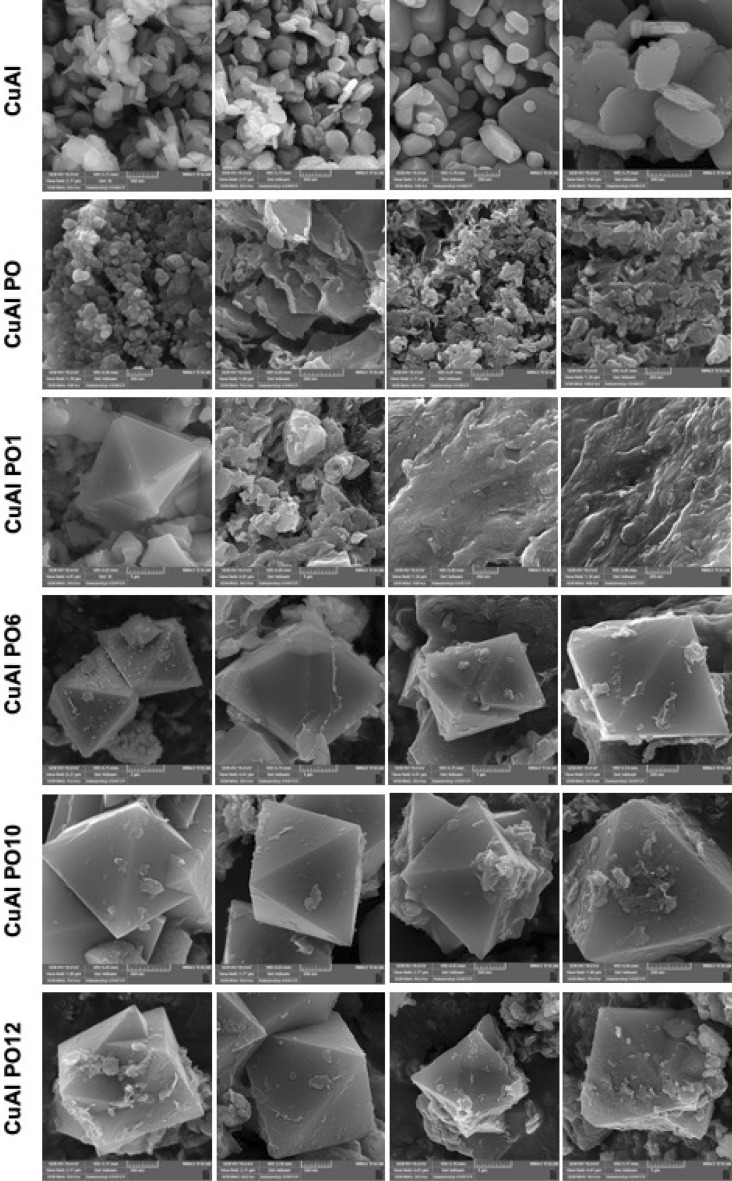
FESEM images of the of the CuAl LDH, CuAl PO LDH, CuAl PO1 LDH, CuAl PO6 LDH, CuAl PO10 LDH and CuAl PO12 LDH.

The FTIR spectra showed that the small molecules (benzamide-like molecules) were synthesized successfully (Fig. 1A,B); FTIR spectra are in complete agreement with our previous study^40^. The strong and wide peak observed for all the samples (both Cu–Al LDH and benzamide-like small molecules) at approximately 3400 to 3500 cm^−1^ are represented to the stretching mode of single bond-OH in the brucite-like layers. In the Cu–Al LDHs, the peak around 1630 cm^−1^ assigned to the interlayered adsorbed/absorbed water molecules, and its weak bending mode appeared at around 3000 cm^−1^. Regarding the crystallinity investigations, the XRD results showed the crystallinity was not changed after coating the leaf extracts (PO) and also incorporating the benzamide-like ligands on the surface; therefore, only a single XRD pattern after surface decoration was conducted (Fig. 2). Besides, all of the XRD patterns showed standard diffractions at 10.2°, 12.8°, 20.5°, 25.8°, 32.2°, 33.6°, 40°, 43.5°, 53° and 61° corresponding to the (003), (006), (009), (012), (015), and (110), in which is in good agreement with the JCPDC 22-0700. Interestingly, the surface morphology of the Cu–Al LDH is without considerable aggregations even after a cost-effective and environmentally-friendly synthesis method (Fig. 1C). However, by surface decoration of the leaf extracts and also the small molecules, some minor aggregations have been observed. These aggregations are because of the hydrogen bonding, van der Waals and other types of chemical/physical interactions between the functional groups of the leaf extracts as well as the small molecules.

The FESEM images (Fig. 2) showed that by decorating the leaf extracts on the surface, the aggregations increased up to a new level, enhanced by the ratio of 60%, however, these aggregations did not have changed by decorating the benzamide-like molecules. Therefore, it could be able to predict that these physical/chemical aggregations/interactions do not have any significant drawbacks in the biomedical applications. By incorporating the small molecules on the surface of the Cu–Al LDH, the crystallinity as well as the bulk morphology of the Cu–Al LDH had not changed, however, the surface functionality of the Cu–Al LDH changes to a new range of fully functional groups. These groups can be able to make new types of interactions as well as the bonds/binds to the guest molecules. Therefore, using these small molecules on the surface of the Cu–Al LDH could be able to direct new types of studies in the biomedical applications.

### Drug release profile

**Figure 3:**
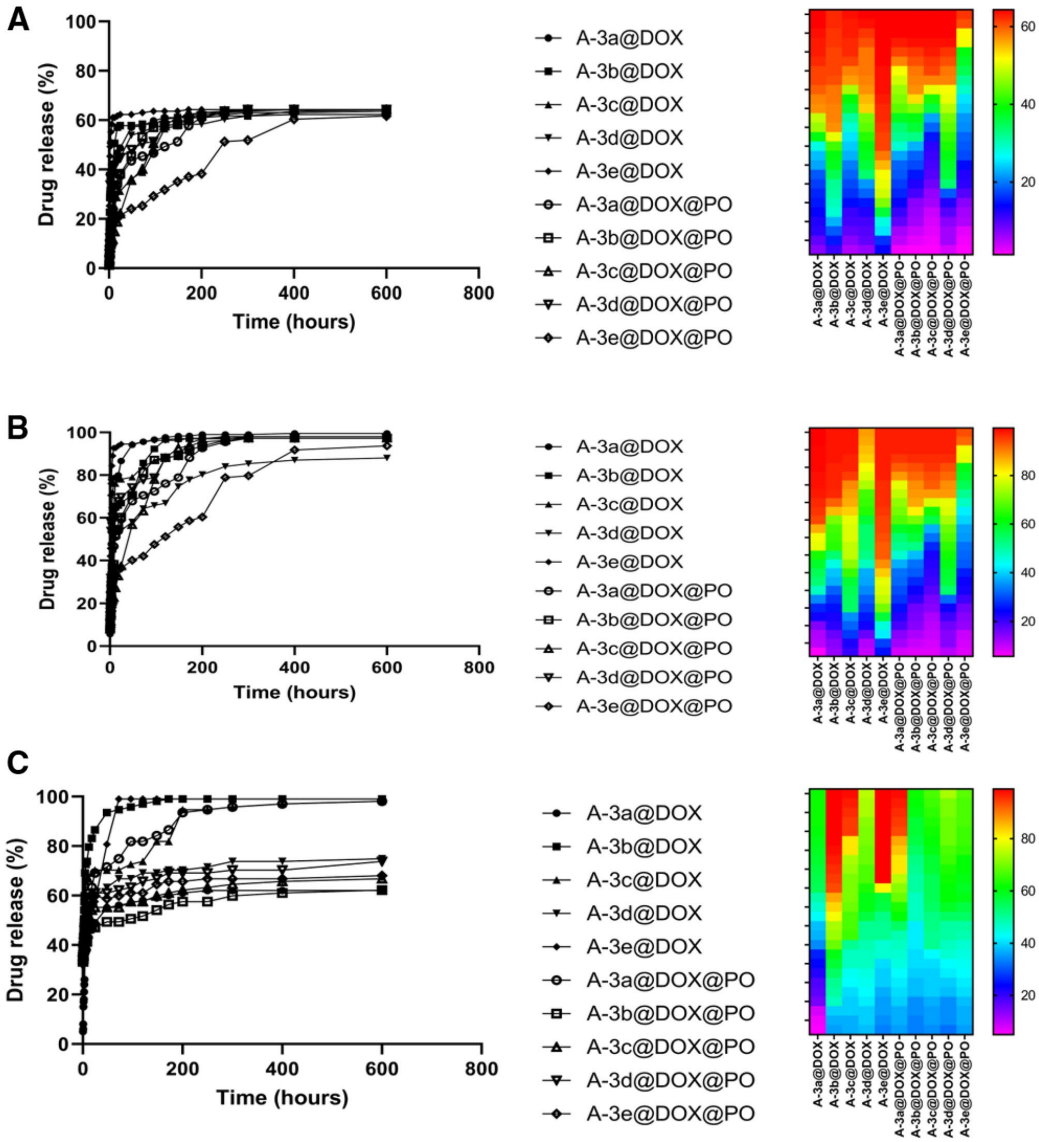
Drug release at pH 4.5 (**A**), pH 5.5 (**B**) and pH 7.2 (**C**). The modification of nanoparticles with PO promotes drug release capacity and they provided sustained release of DOX. The DOX release was steady after 250 h. Highest release occurred at pH 5.5 and lowest release was observed at pH 4.5.

The tumor microenvironment (TME) has a mild acidic pH that is due to rapid proliferation of cancer cells. The tumor cells use glycolysis instead of oxidative phosphorylation to meet their needs to energy. Use of glycolysis for energy production leads to generation of pyruvic acid from glucose, leading to significant decrease in pH level^41^. Such acidic pH can be utilized for development of nanostructures that are pH-sensitive and can release cargo at tumor site. Recently, various kinds of nanostructures have been designed for delivery of DOX that are pH-sensitive. In an effort, pH-sensitive albumin nanoarchitectures were developed for DOX delivery in lung cancer suppression. These nanoparticles released DOX at a pH lower than physiological pH and this promoted accumulation of anti-tumor agent in tumor site, resulting in apoptosis^42^. Another study synthesized pH-sensitive polymerosomes for DOX delivery and glioblastoma treatment. This experiment evaluated drug release at different pH levels of 5.5, 6.5 and 7.4. The results revealed that polymerosomes released more DOX at pH 5.5 compared to 6.5 and 7.4 that is similar to pH of TME^43^. Furthermore, DOX-loaded pH-sensitive lipid nanocarriers were synthesized in breast cancer suppression. This study evaluated DOX release from lipid nanostructures at pH of 5.0, 6.8 and 7.4. The four-time increase occurred in DOX release at pH 5.0 compared to pH 7.4^44^. Another study synthesized PEGylated pH-sensitive nanogels for DOX delivery. The highest release of DOX occurred in pH 5.0, while lowest release occurred in pH 7.4, showing pH-sensitivity of nanogels and their promising application for purpose of cancer therapy^45^. In the present study, we also showed that more drug release occurs in pH 4.5 compared to pH 7.2. We evaluated release of DOX from nanostructures at three different pH levels including pH 4.5, pH 5.5 and pH 7.2 (Fig. 3). It was found that DOX release from green synthesized LDH nanoparticles enhances by time (steady after 250 h). The lowest drug release occurred in pH 4.5 and maximum 60% release of DOX was observed. Highest drug release occurred in pH 5.5 and in some cases, it reached 90%. The drug release also occurred in pH 7.2, but it was lower than pH 5.5 and higher than pH 4.5. Furthermore, it was found that DOX release was higher in PO-modified LDH nanoparticles compared to non-modified LDH nanostructures. According to results from drug release at pH levels of 5.5 and 7.2, it seems that PO-modified LDH nanoparticles are stable at acidic pH levels and they can also provide DOX release for purpose of cancer therapy. Noteworthy, as drug release reduces at pH 4.5, it can be concluded that some changes in stability and structure of LDH nanoparticles may occur in pH 4.5. However, as pH of TME is at the range of 5.5–7, it can be concluded that PO-modified LDH nanoparticles are ideal candidates for DOX release in cancer suppression. Also, by increasing the push–pull electronic effect on the benzamide-core-based linkers anchored on the surface of the LDHs, the drug release manipulated to more sustained profile. Therefore, the results of this study showed slow and controlled drug release of DOX up to 600 h.

### Antibacterial activity

The antimicrobial agents are extensively utilized for management and treatment of infections. Various kinds of antibacterial agents including natural, synthetic and semi-synthetic have been designed in treatment of bacterial infections. However, drug resistance in bacteria has provided a challenge in treatment of infections and efforts have been made in developing novel therapeutics for bacterial infections^46–48^. Nanotechnology has opened a new gate in treatment of bacterial infections. Recently, caffeine-loaded gold nanostructures were developed for treatment of bacterial infections. These nanoarchitectures demonstrated potential in suppressing biofilm generation, dispersing mature biofilms and elimination of Gram-positive and -negative bacteria^49^. In another effort, selenium nanoparticles were prepared for evaluating their cytotoxicity against multidrug resistance (MDR) bacteria. At concentrations of 20, 80, 320, and > 320 μg/mL, selenium nanostructures effectively demonstrated cytotoxicity against MDR bacteria and they also showed synergistic impact in combination therapy with linezolid as a conventional antibiotic^50^. Therefore, nanostructures can be employed for elimination, inactivation and sterilization purposes and bacteria cannot develop resistance to these cytotoxic agents^51,52^.

**Figure 4:**
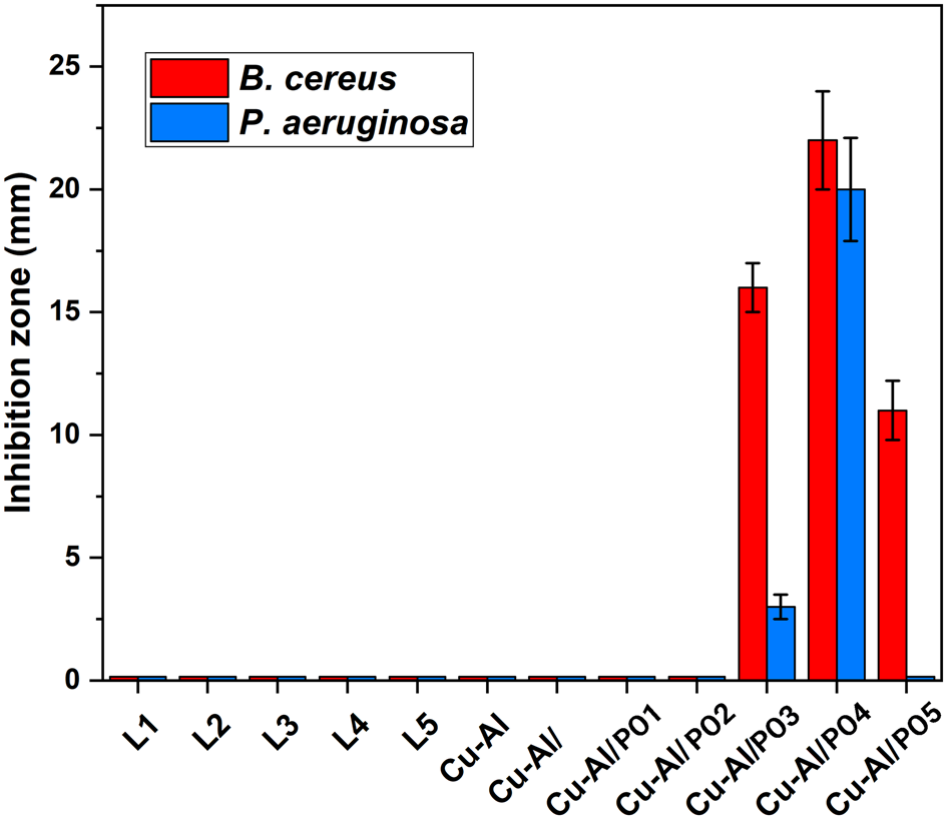
Antibacterial activity of the samples against *Bacillus cereus* and *Pseudomonas aeruginosa* at 5 mg/mL concentreation.

The agar diffusion disc method was used to investigate the antibacterial activity of CuAl, CuAl/PO, CuAl/PO1, CuAl/PO2, CuAl/PO3, CuAl/PO4, CuAl/PO5, L1, L2, L3, L4 and L5 in different concentrations of 50 mg/mL, 5 mg/mL, 0.5 mg/mL, 0.05 mg/mL, and 0.005 mg/mL on both Gram-negative *Pseudomonas aeruginosa* and Gram-positive *Bacillus cereus* bacteria within 24 h (Fig. 4). Mueller–Hinton agar was used as the culture medium for the bacteria. The bacteria (1.5 × 10^8^ cells/mL) were seeded by dispersing the culture medium containing the bacteria on the surface of nutrient agar plates and afterward, disks were soaked in 0.005–50 mg/mL of the NP’s suspensions and positioned on the injected agars and incubated for 24 h at 37 °C. A clear zone of inhibition (IZD) was distinguished and reported after the incubation.

As can be seen, samples L1 to L5, Cu–Al, Cu–Al/PO to Cu–Al/PO2 did not show antibacterial effect against the tested microorganisms. Cu–Al/PO3 and Cu–Al/PO4 displayed antibacterial properties toward both bacteria while Cu–Al/PO5 only demonstrated activity against *B. cereus*. Among the samples, Cu–Al/PO4 exhibited the highest antibacterial activity. In addition, the samples possessing antibacterial activity showed higher inhibition zone against the gram-positive bacterium (*B. cereus*), compared to gram-negative bacterium (*P. aeruginosa*).

### Cell viability

**Figure 5:**
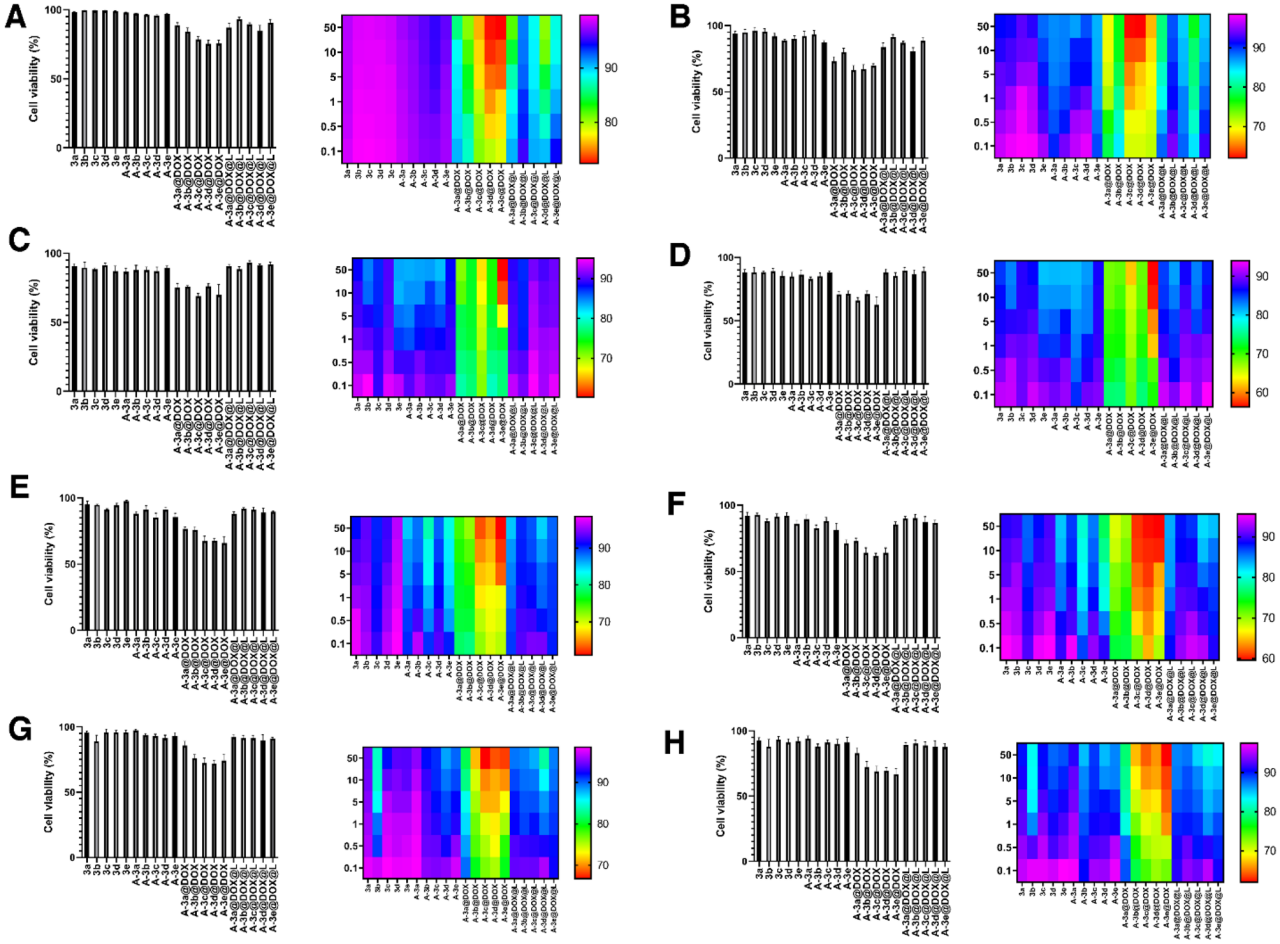
The MTT assay for HEK-293 cells after 24 h (**A**) and 48 h (**B**); HT-29 cells after 24 h (**C**) and 48 h (**D**); MCF-7 cells after 24 h (**E**) and 48 h (**F**); PC12 cells after 24 h (**G**) and 48 h (**H**). The heat-map profile images showed concentration (dose)-dependent results from 0.1 to 50 μg/mL.

In recent decades, green synthesis of nanoparticles has obtained special attention for improving biocompatibility and safety profile. A recent experiment attempted to synthesis chromium oxide nanoparticles from leaf extract of *Abutilon indicum* and they showed higher biocompatibility compared to chemically synthesized nanostructures^53^. Another experiment used *Cistus incanus* for green synthesis of silver nanoparticles and resulting nanocarriers were spherical in shape with particle size of 68.8–71.2 nm. The biocompatibility of silver nanostructures on fibroblasts was examined and they showed high cytobiocompatibility. Furthermore, green synthesized silver nanostructures did not show monocyte activation and had immuno-compatibility feature^54^. The green synthesized nanoparticles are of interest in field of cancer chemotherapy to minimize cytotoxicity on normal cells. An experiment used extract of *Peltophorum pterocarpum* (PP) leaves for green synthesis of gold nanoparticles and then, DOX was loaded on nanostructures. The results of study demonstrated that DOX-loaded green synthesized gold nanoparticles have high biocompatibility and no remarkable alteration occurs in hematology, biochemistry and histopathology of mice after intraperitoneal injection^55^. Furthermore, synthesis of nanoparticles with naturally occurring compounds is a cost-effective and eco-friendly process. The green synthesized nanoparticles have potential to be used for delivery of chemotherapeutic agents such as DOX and their large-scale production is also possible^56^. Hence, our experiment focused on designing LDH nanoparticles and their green synthesis by PO to improve their biocompatibility and make the process cost-effective. The MTT assay was performed to evaluate biocompatibility of PO-modified LDH nanoparticles. The HEK-293 and PC12 cells were chosen as normal cells, and HT-29 and MCF-7 cells were selected as cancer cells. The Fig. 5A,B demonstrates impact of DOX-loaded PO-modified LDH nanoparticles on HEK-293 cells and based on the results, DOX-loaded nanoparticles show high safety profile. Exposure of HEK-293 cells to DOX-loaded LDH nanostructures leads to a decrease in cell viability; however, DOX-loaded PO-modified LDH nanostructures show high biocompatibility compared to non-modified LDH nanoparticles, displaying that presence of PO can be beneficial in improving biocompatibility. The Fig. 5G,H is also about biocompatibility of DOX-loaded PO-modified LDH nanoparticles on PC12 cells. Similarly, PO-modified LDH nanoparticles demonstrate negligible toxicity on PC12 cells, confirming their high biocompatibility. Two kinds of tumor cells including colon and breast cancer cells were chosen to evaluate toxicity of prepared nanoparticles (Fig. 5C–F). The DOX-loaded LDH nanoparticles reduced viability of HT-29 and MCF-7 cells, while the decrease in cell viability was lower in PO-modified LDH nanostructures. Therefore, modification and green synthesis of LDH nanoparticles with PO can significantly enhance biocompatibility of these nanostructures.

**Figure 6:**
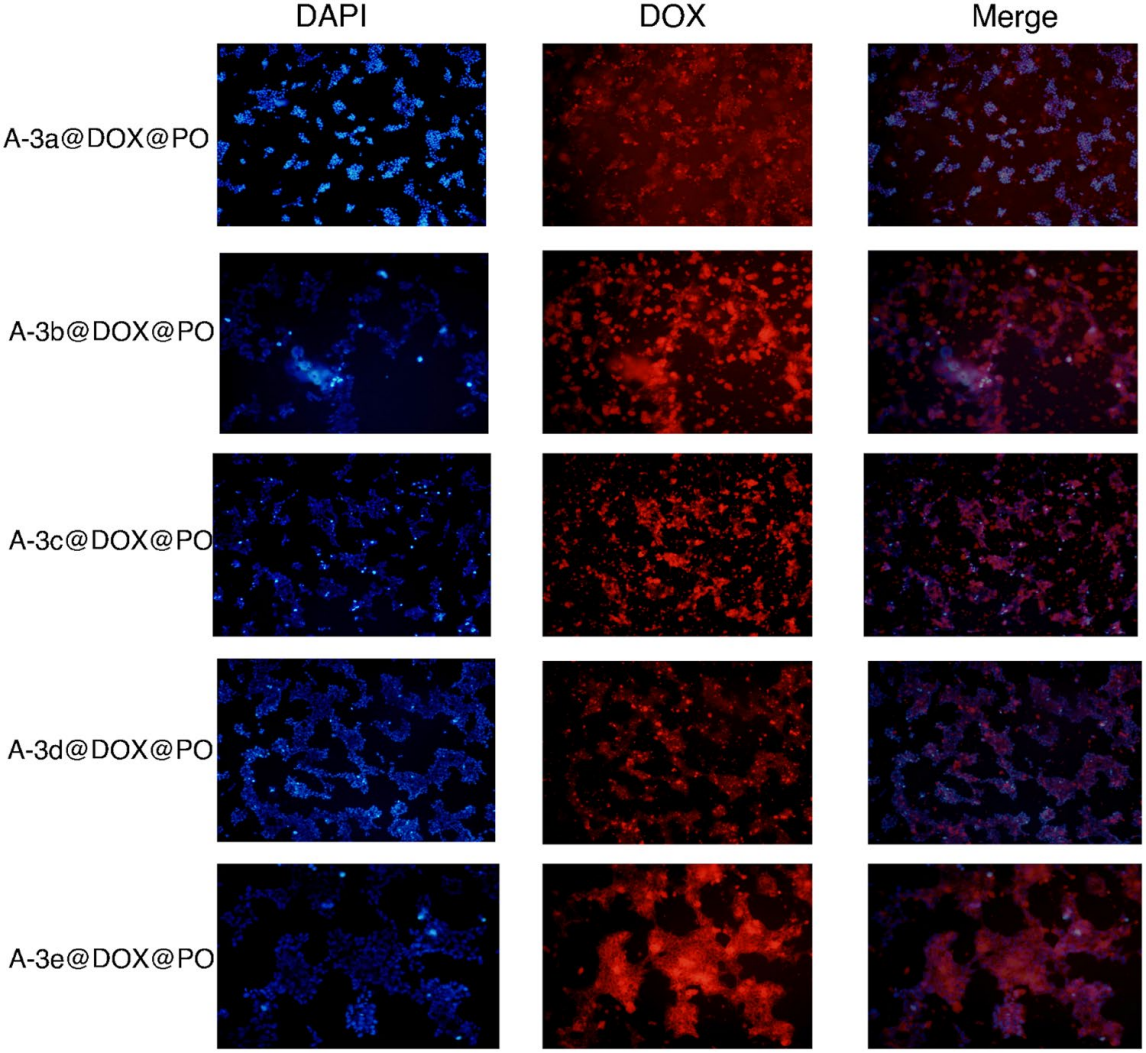
The CLSM images of the drug loaded nanocarriers-coated with leaf extracts treated with HEK-293 cell lines. The used concentration of the nanoparticles: 17.5 μg/mL. The scale bar is same as the Fig. 7.

**Figure 7:**
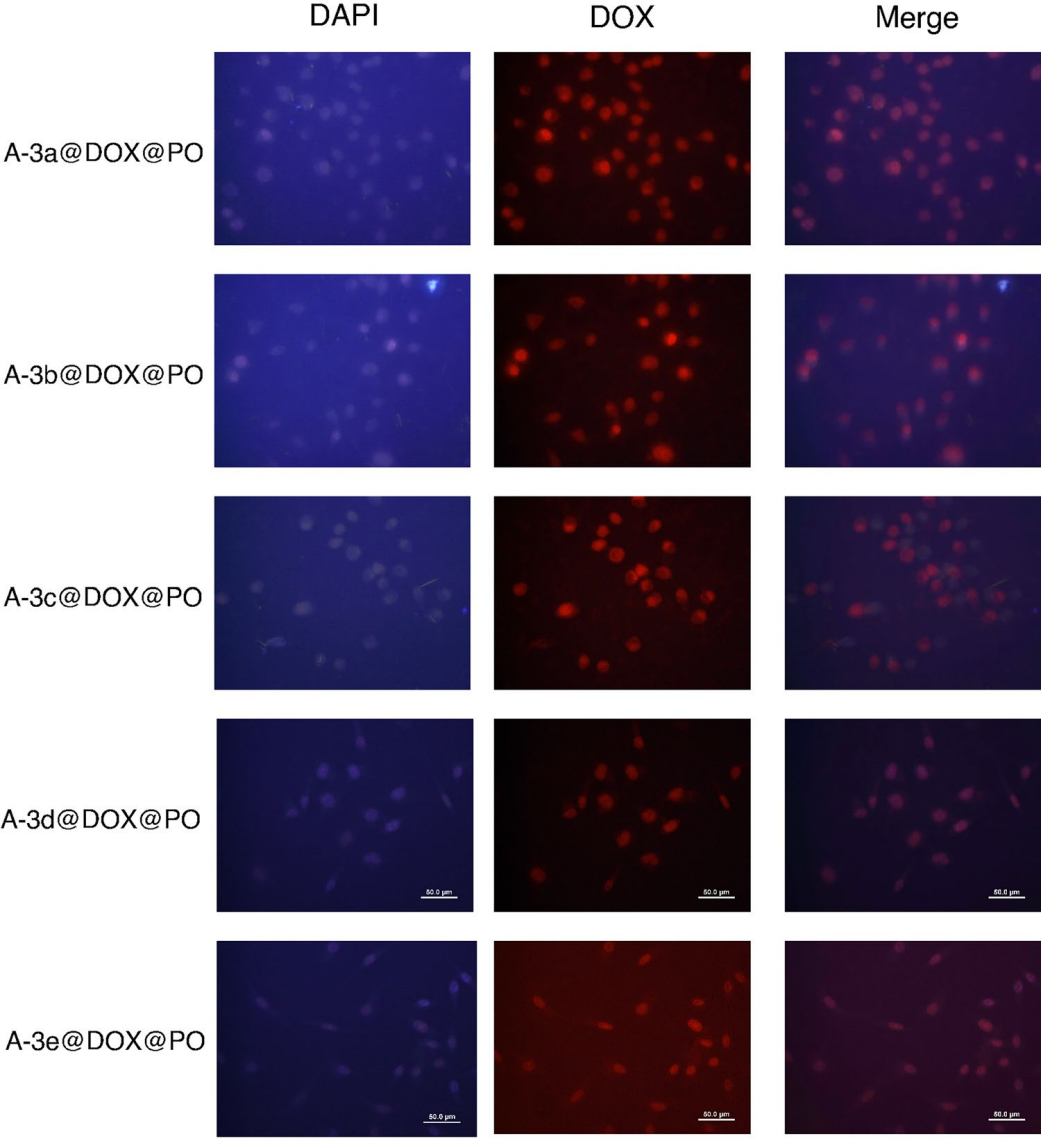
The CLSM images of the drug loaded nanocarriers-coated with leaf extracts treated with MCF-7 cell lines. The used concentration of the nanoparticles: 17.5 μg/mL.

The previous results revealed pH-sensitive release of DOX from PO-modified Cu–Al LDH nanostructures and also, their high biocompatibility demonstrated by MTT assay. In order to further approve potential of LDH nanostructures for DOX delivery, we examined their interaction with HEK-293 and MCF-7 cells (Figs. 6, 7). The Fig. 6 reveals presence of nanocarriers in cytoplasm and nuclei of HEK-293 cells that we can also observe in Fig. 7 for MCF-7 cells. Therefore, cellular uptake of DOX significantly enhances using LDH nanostructures and they can provide nuclear delivery of this anticancer agent that is of importance for improving its action on suppressing the activity of topoisomerase II enzymes and preventing DNA replication. Furthermore, Fig. 6 shows that structure and morphology of HEK-293 cells as normal cells have not been changed by DOX-loaded PO-modified Cu–Al LDH nanostructures, further confirming high biocompatibility and safety profile of nanoparticles.

**Figure 8:**
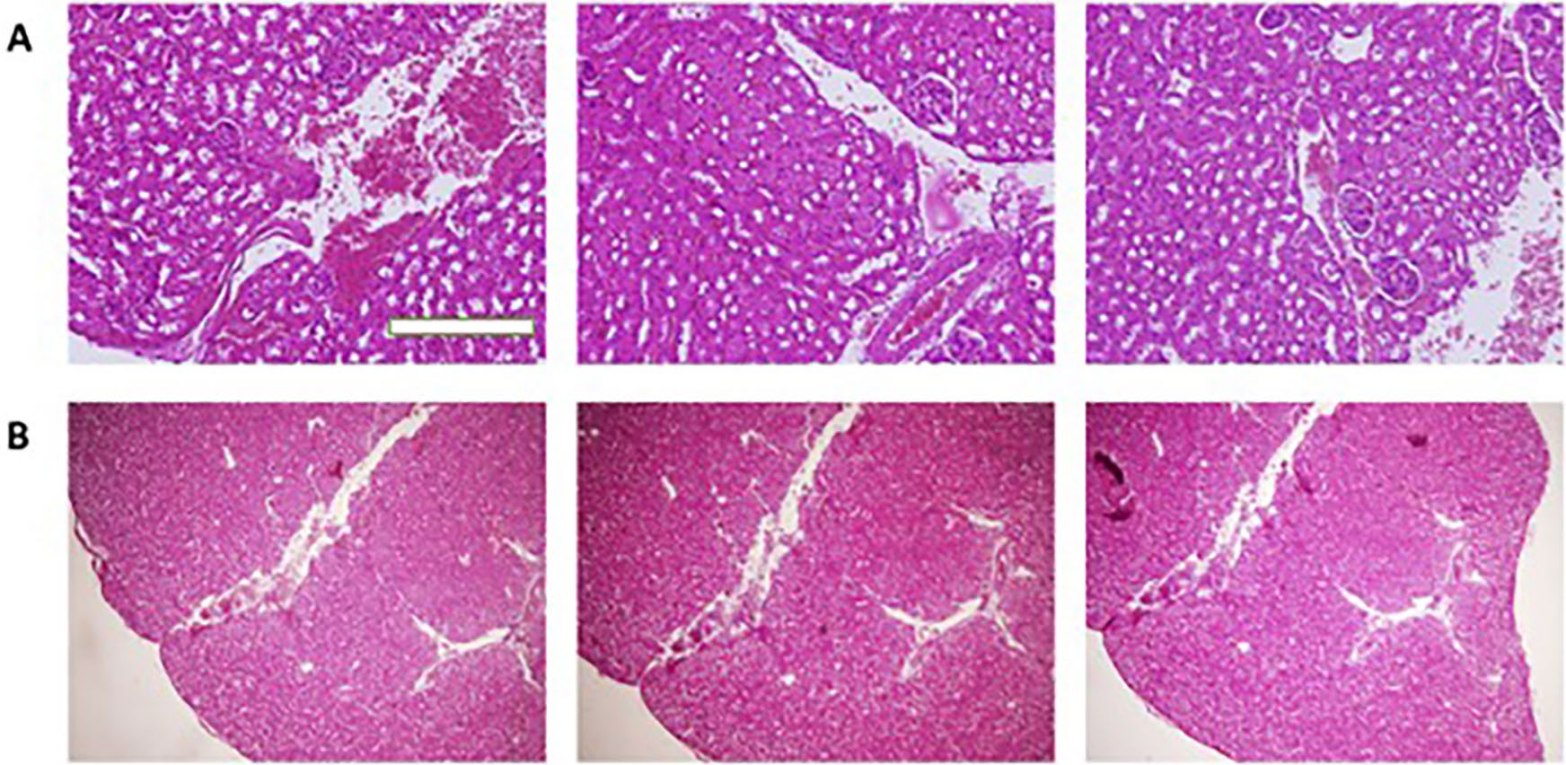
The histopathological analysis of kidney tissue upon application of DOX-loaded nanostructures. The morphology of cells in tissue has not changed and no vessel occlusion is observed, showing high biocompatibility of nanoparticles (**A,B**). The used concentration of the nanoparticles: 17.5 μg/mL. The scale bar is 50 μm.

Although LDH nanostructures are extensively employed in various fields from industrial catalysis to pharmaceuticals, evaluating their biocompatibility is of great important to prevent unexpected side effects. A number of studies have shown toxicity of LDH nanoparticles that may result from their capacity in promoting reactive oxygen species (ROS) generation, mediating oxidative stress and elevating lipid peroxidation^57^. It has been reported that LDH nanoparticles have high safety profile at range of 10–500 µg/mL, so that at concentration of 10 µg/mL, LDH nanoparticles do not affect viability of normal cells. At concentration of 500 µg/mL, they have partial impact on RBCs’ lysis and they do not activate complement system in a significant rate^58^. Furthermore, LDH nanostructures can cause systemic toxicity after intravenous administration due to aggregation with erythrocytes. Noteworthy, coating LDH nanostructures with lipid membrane significantly enhances their biocompatibility, prevents aggregation and improves mouse survival after intravenous administration of nanoparticles^59^. Therefore, surface modification of LDH nanoparticles and/or their green synthesis can be considered as promising strategies in improving biocompatibility of these nanocarriers. Based on Fig. 8A,B, we evaluated toxicity and safety profile of PO-modified DOX-loaded LDH nanoparticles on kidney tissue, as one of the major organs for accumulation. As it is shown in Fig. 8, the morphology of cells in kidney tissue has not been changed after LDH nanoparticle administration and no blood occlusion is observed. According to these results, green synthesis of LDH nanoparticles with PO improves the biocompatibility of these nanocarriers and make them safe options for drug delivery applications.

## Conclusion and remarks

The present study used PO for the first time, for surface modification of Cu–Al LDH nanostructures to improve their potential as nano-scale delivery systems. After preparation of nanoparticles, DOX as an anti-cancer drug was loaded onto them and characterization methods confirmed correct synthesis and drug loading. The drug release study revealed pH-sensitive release of DOX from LDH nanoparticles and highest release of anti-tumor drug occurred in pH 5.5 and lowest drug release was related to pH 4.5 that is maybe due to negative impact of low and highly acidic pH on structure of nanoparticles. The MTT assay revealed high biocompatibility of PO-modified Cu–Al LDH nanoparticles; so that PO-modified LDH nanoparticle demonstrated partial and negligible toxicity towards PC12 and HEK-293 cells, while they diminished viability of HT-29 and MCF-7 cells, as tumor cells. Noteworthy, reduction in viability of HT-29 and MCF-7 cells was lower in PO-modified LDH nanoparticles compared to non-modified LDH nanocarriers that its reason should be evaluated and analyzed in next experiments. The CLSM figures showed high cellular uptake, and delivery of DOX by LDH nanoparticles to cytoplasm and nucleus of MCF-7 and HEK-293 cells. Furthermore, histopathological analysis of kidney tissue demonstrated no damage in cells, normal morphology of cells and tubules and no blood vessel occlusion, further confirming the high biocompatibility of PO-modified LDH nanoparticles. The antibacterial test showed that Cu–Al LDH nanostructures have cytotoxicity against Gram-positive and -negative bacteria and they can be utilized in future experiments for treatment of bacterial infections.

## Supplementary Information

Supplementary Information.

## Data Availability

All data generated or analysed during this study are included in this published article and its Supplementary Information files.

## Abbreviations

DOX: Doxorubicin
LDH: Layered double hydroxide
PO: *Plantago ovate*
TME: Tumor microenvironment
PP: *Pletophorum pterocarpum*
