# Supplementary material for: Multifunctional green synthesized Cu–Al layered double hydroxide (LDH) nanoparticles: anti-cancer and antibacterial activities
Source: Sci Rep. 2022 Jun 8;12:9461. doi: 10.1038/s41598-022-13431-7 (PMC9177833; doi:10.1038/s41598-022-13431-7)
Supplement: Supplementary file 1 — Supplementary Information. [file 41598_2022_13431_MOESM1_ESM.docx]

Supporting Information

**Multifunctional green synthesized Cu-Al layered double hydroxide (LDH) nanoparticles: Anti-cancer and antibacterial activities**

Mahsa Kiani^1^, Mojtaba Bagherzadeh^1,*^, Amir Mohammad Ghadiri^1^, Pooyan Makvandi^2^, Navid Rabiee^1,3^

1. Department of Chemistry, Sharif University of Technology, Tehran, Iran
2. Istituto Italiano di Tecnologia, Center for Materials Interfaces, viale Rinaldo Piaggio 34, 56025 Pontedera, Pisa, Italy
3. School of Engineering, Macquarie University, Sydney, New South Wales, 2109, Australia

*Corresponding author: Mojtaba Bagherzadeh ([bagherzadeh@sharif.edu](mailto:bagherzadeh@sharif.edu))

## Green synthesis of molecules

Multi-step synthesis of molecules was performed by mechanochemical and standard reactions (Scheme 1); the objective is to enhance the biocompatibility of the final products, along with significantly reducing the generation of pollutants.

Scheme 1. Synthesis procedure of the deployed molecules.

**4-aminobenzohydrazide (2)**

Benzocaine (**1)** (5 mmol) and hydrazine hydrate (20 mmol) were melted together (115 °C, 60 min) in a closed container. Recrystallization was conducted by using EtOH yielded needle crystalline product. Yield 85%, m.p: 321-324 °C, IR (KBr) ν (cm^-1^) 3300-3200 (NH, NH_2_), 1670, 1650 (C=O), LC-MS (ESI) m/z 256 (M+H).

**4-(2-(4-(3-carboxypropanamido)benzoyl)hydrazineyl)-4-oxobutanoic acid (3a)**

*p*-Amino benzohydrazide (**2)** (0.151g, 1 mmol) with succinic anhydride (2.02 mmol) was refluxed in ethyl acetate (20 mL) for 12 hours. The organic solvent was removed under vacuum and the mixture was poured into 30 mL of distilled water and stirred for 30 minutes. The reaction was made acidic by adding concentrated HCl. The crude product was dried and then recrystallized from EtOAc and finally purified by a short column of silica gel and mobile phase of hexane: chloroform (20:80). Yield 45%, m.p: 185-187 °C. IR (KBr) ν (cm^-1^) 3200-2800 (NH, COOH), 2750-2600 (-CH_2_-CH_2_), 1700 (C=O), 1200-1400 (Ar). ^1^H NMR(400 MHz, DMSO) δ*ppm* 11.66 (s, 2H, 2 COOH), 10.32-10.23 (d, 3H, 3NH), 7.80 (d, *J* = 8.5 Hz, 2H, Ar), 7.67 (d, *J* = 8.5 Hz, 2H, Ar), 2.60 (d, *J* = 6.3 Hz, 2H, -CH_2_), 2.55 (d, *J* = 6.1 Hz, 2H, -CH_2_), 1.99-1.94 (d, 4H, -CH_2_). ^13^C NMR (101 MHz, DMSO) δ*ppm* 174.30, 171.00, 163.11, 160.17, 142.45, 129.03, 128.56, 118.45, 31.56, 29.12, 25.56, 18.45. LC-MS (ESI) m/z 350 (M-H). Anal. Calcd. for C_15_H_17_N_3_O_7_: C, 51.28; H, 4.88; N, 11.96. Found: C, 51.20; H, 4.91; N, 12.04.

**General procedure for the green synthesis of the compounds 3b-3e**

*p*-Amino-benzohydrazide (**2)** (0.151g, 1 mmol) with the either acyl chloride or benzenesulfonyl chloride (2.02 mmol) was stirred in pyridine (5 ml) for 24 hours. The reaction mixture was poured into 30 mL of distilled water and stirred for 15 minutes. The pH of the mixture was decreased by adding concentrated HCl. The precipitate was then recrystallized from EtOH to afford white amorphous solid.

**4-fluoro-N-(4-(2-(4-fluorobenzoyl)hydrazine-1-carbonyl)phenyl)benzamide (3b)**

Yield: 60%, m.p: 322-325 °C, IR (KBr) ν (cm^-1^) 3700-3100 (NH), 1700-1650 (C=O), 1200-1400 (Ar). ^1^H NMR(400 MHz, DMSO) δppm 10.55 (s, 2H, 2NH), 10.51 – 10.46 (m, 1H, NH), 8.05 (dd, J = 8.4, 5.4 Hz, 4H, Ar), 7.98 – 7.89 (m, 4H, Ar), 7.39 (d, J = 8.7 Hz, 4H, Ar). ^13^C NMR (101 MHz, DMSO) δppm 165.93, 165.81, 165.32, 165.26, 163.45, 142.80, 131.55, 131.09, 131.00, 130.69, 130.60, 128.75, 127.81, 120.06, 116.13, 116.01, 115.92, 115.79. LC-MS (ESI) m/z 394 (M-H). Anal. Calcd. for C_21_H_15_F_2_N_3_O_3_: C, 63.80; H, 3.82; N, 10.63. Found: C, 63.74; H, 3.86; N, 10.61.

**4-methoxy-N-(4-(2-(4-methoxybenzoyl)hydrazine-1-carbonyl)phenyl)benzamide (3c)**

Yield: 67%, m.p: 264-266 °C, IR (KBr) ν (cm^-1^) 3300-3100 (NH), 1700-1650 (C=O), 1200-1400 (Ar). ^1^H NMR(400 MHz, DMSO) δppm 10.36 (s, 3H, 3NH), 7.99 (d, J = 8.8 Hz, 2H, Ar), 7.92 (d, J = 5.5 Hz, 6H, Ar), 7.07 (m, 4H, Ar), 3.85 (s, 3H, OMe), 3.84 (s, 3H, OMe). ^13^C NMR (101 MHz, DMSO) δppm 165.89, 165.69, 162.58, 162.47, 143.04, 130.24, 128.68, 127.62, 127.10, 125.22, 119.93, 114.19, 114.15, 55.94, 55.87. LC-MS (ESI) m/z 418 (M-H). Anal. Calcd. for C_23_H_21_N_3_O_5_: C, 65.86; H, 5.05; N, 10.02. Found: C, 65.94; H, 5.02; N, 10.00.

**4-nitro-N-(4-(2-(4-nitrobenzoyl)hydrazine-1-carbonyl)phenyl)benzamide (3d)**

Yield: 74%, m.p: 287-289 °C, IR (KBr) ν (cm^-1^) 3300-3100 (NH), 1700-1650 (C=O), 1350, 1550 (NO), 1200-1400 (Ar). ^1^H NMR (400 MHz, DMSO) δppm 10.86 (s, 1H, NH), 10.72 (s, 2H, 2NH), 8.40 (m, 4H, Ar), 8.26 – 8.15 (m, 4H, Ar), 8.02 – 7.85 (m, 4H, Ar). ^13^C NMR (101 MHz, DMSO) δppm 165.53, 164.76, 149.83, 149.75, 142.45, 140.77, 138.85, 129.83, 129.44, 128.84, 128.17, 124.25, 124.08, 120.25. LC-MS (ESI) m/z 448 (M-H). Anal. Calcd. for C_21_H_15_N_5_O_7_: C, 56.13; H, 3.36; N, 15.58. Found: C, 56.09; H, 3.39; N, 15.55.

**N-(4-(2-(phenylsulfonyl)hydrazine-1-carbonyl)phenyl)benzenesulfonamide (3e)**

Yield: 38%, m.p: 239-241 °C, IR (KBr) ν (cm^-1^) 3700-3300 (NH), 1700-1650 (C=O), 1200-1400 (Ar), 1100-1200 (SO_2_). ^1^H NMR (400 MHz, DMSO) δppm 11.40 (s, 1H, NH), 10.88 (s, 2H, 2NH), 7.97– 7.50 (m, 14H, Ar). ^13^C NMR (101 MHz, DMSO) δppm 166.03, 142.33, 139.68, 138.63, 135.10, 133.76, 129.94, 129.67, 129.24, 128.97, 128.05, 127.14, 126.29, 118.65. LC-MS (ESI) m/z 430 (M-H). Anal. Calcd. for C_19_H_17_N_3_O_5_S_2_: C, 52.89; H, 3.97; N, 9.74. Found: C, 52.80; H, 3.94; N, 9.78.
